# Sensorimotor Integration and Pain Perception: Mechanisms Integrating Nociceptive Processing. A Systematic Review and ALE-Meta Analysis

**DOI:** 10.3389/fnint.2022.931292

**Published:** 2022-08-05

**Authors:** Cindy Gombaut, Scott A. Holmes

**Affiliations:** ^1^Pediatric Pain Pathway Lab, Department of Anesthesia, Critical Care, and Pain Medicine, Boston Children's Hospital, Boston, MA, United States; ^2^Department of Anesthesia, Harvard Medical School, Boston, MA, United States

**Keywords:** sensorimotor integration (SMI), motor system, chronic pain, sensorimotor processing and motor diseases, pain perception, nociceptive processing, fMRI

## Abstract

Pain treatment services and clinical indicators of pain chronicity focus on afferent nociceptive projections and psychological markers of pain perception with little focus on motor processes. Research supports a strong role for the motor system both in terms of pain related disability and in descending pain modulation. However, there is little understanding of the neurological regions implicated in pain-motor interactions and how the motor and sensory systems interact under conditions of pain. We performed an ALE meta-analysis on two clinical cohorts with atypical sensory and motor processes under conditions of pain and no pain. Persons with sensory altered processing (SAP) and no pain presented with greater activity in the precentral and supplementary motor area relative to persons with self-reported pain. In persons with motor altered processing (MAP), there appeared to be a suppression of activity in key pain regions such as the insula, thalamus, and postcentral gyrus. As such, activation within the motor system may play a critical role in dampening pain symptoms in persons with SAP, and in suppressing activity in key pain regions of the brain in persons with MAP. Future research endeavors should focus on understanding how sensory and motor processes interact both to understand disability and discover new treatment avenues.

## Introduction

The motor system has a poorly understoodd role in nociceptive processing and pain perception. The relationship between nociception and pain is largely understood as sensory in origin; however, acute and chronic pain is associated with significant physical disability (Dudgeon et al., 2002) and motor-inhibitory processes (Le Pera et al., 2001) that implicate the motor system as both a downstream and up-stream effector on pain perception. The prevalence of chronic pain is roughly 20.4% of the US population with ~8.0% having high-impact chronic pain; meaning chronic pain that limits life or work activities (Dahlhamer, 2018). To date, it remains unclear how the motor and sensory regions of the central nervous system are impacted relative to each other under conditions of pain.

The primary motor and sensory cortex share reciprocal efferent and afferent pathways. Online motor performance has been shown to modulate sensory processing, both prior to and during active movement (Angel and Malenka, 1982; Starr and Cohen, 1985; Jiang et al., 1990; Buckingham et al., 2010; Seki and Fetz, 2012; Juravle et al., 2017; Fraser and Fiehler, 2018; Voudouris et al., 2019). Physical activity programs have reliably shown a beneficial effect for persons suffering from chronic pain conditions (Ambrose and Golightly, 2015; Daenen et al., 2015; Booth et al., 2017), where motor activity (Hautasaari et al., 2020) and motor imagery (Larsen et al., 2019) directly influence cortical activity during active nociceptive stimulation. This connection may be mediated in part by direct efferent pathways connecting the primary motor and sensory cortex through feed forward and feedback processes (Umeda et al., 2019), or through long range efferent pathways that integrate the peripheral nervous system. It is still not clear how nociception and pain processing interfere with canonical sensorimotor processing.

Short and long-range connections between sensory and motor regions of the brain may be implicated in the processing of nociceptive stimuli and pain perception. Incongruence between *efferent* pathways from the motor cortex with the *afferent* feedback to the primary sensory cortex through indirect connections that integrate peripheral nerve and muscles may underlie chronic pain. We evaluated clinical populations with altered motor and sensory feedback based on (1) the relatively lower prevalence of pain in persons with motor altered processing (MAP) than sensory altered processing (SAP), (2) the connection between motor and descending pain modulation areas of the brain, and (3) sensorimotor incongruence subserving learning and long-term potentiation. We predicted that altered afferent feedback, seen in persons with SAP, would be associated with aberrant processing in motor planning areas of the brain and that atypical efferent activity, seen in persons with MAP, would be associated with activation in pain-related regions of the brain.

## Methods

### Database Search

We conducted a systematic review of the literature according to the updated Preferred Reporting Items for Systematic Reviews and Meta-Analyses (PRISMA) 2020 guidelines (Page et al., 2021) and checklist (see Supplementary Material S1). Searches were performed in the following databases presented with their respective timelines: Pubmed (1950–2021) and Google Scholar (1950–2021). Database searches were organized according to: *Populations, Neuroimaging Methods*, and *Task Specification*. These search terms were combined using the operator “AND” reflecting between parameter combinations and “OR” reflecting within parameter searches. If the Pubmed database was being used, key words were first searched through the Pubmed MeSH database to include additional subheadings or quantifiers within the same context of the key word. See **Figure 2** for an example of the search methodology. *Population* key terms (see Table 1) searched were “*spinal cord injury*,” “*SCI*,” “*amputees*,” “*phantom limb pain*,” “*pain*.” *Neuroimaging Methods* key terms searched were “*MRI*,” “*fMRI*,” “*task-based fMRI*,” “*cerebral activation*.” *Task Specification* key terms searched were “*movement execution*,” “*movement imagery*.” Manual searches were completed through the reference lists of the included articles. All studies that met the inclusion criteria were reviewed in full whereas others were reviewed solely by abstract. One reviewer screened each record independently and the second reviewer screened the studies that met the inclusion criteria for data extraction.

**Table 1:** List of included sensory and motor disorders.

**Sensory disorders**		**Motor disorders**	
Spinal cord injury		Dystonia	
	Complete thoracic SCI; complete lumbar SCI		Cervical dystonia; focal upper limb dystonia; generalized idiopathic torsion dystonia; multifocal idiopathic torsion dystonia
Amputees	Unilateral upper limb amputees; bilateral upper limb amputees; unilateral lower limb amputees	Parkinson's disease	Probable PD; akinetic-rigid PD; tremor-dominant PD; mixed type PD; PD with freezing of gait

**Figure 1:**
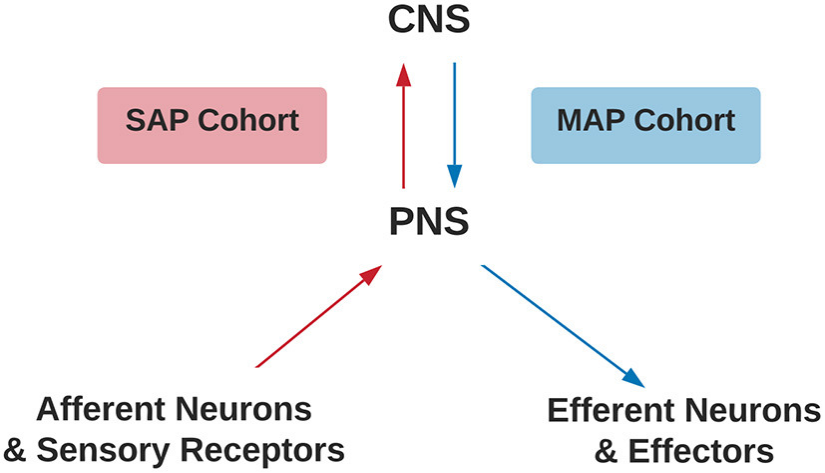
Criteria for SAP and MAP cohorts. Assignment of SAP (red) or MAP (blue) cohorts differentiated by disruption to afferent or efferent CNS processing, respectively. CNS, Central Nervous System; PNS, Peripheral Nervous System; SAP, Sensory Altered Processing; MAP, Motor Altered Processing.

The inclusion criteria were the study provided stereotaxic coordinates of cortical activity averaged within-group comparisons or a single representative subject during a movement imagery or movement execution task in the SAP and MAP cohorts listed in Table 1 with or without pain. Disruptions to afferent pathways and processing in the CNS were grouped into the SAP cohort (see Figure 1). Criteria for SAP included complete spinal cord injuries defined as loss of sensory and motor function below the point of injury and amputees defined as amputation of all or part of an arm or leg. Disruptions to efferent pathways and processing in the CNS were grouped into the MAP cohort (see Figure 1). Criteria for MAP included (1) Dystonia: characterized by involuntary and sustained muscle contractions leading to twisting, repetitive movements and abnormal postures (*Dystonia—Symptoms and Causes*, 2020) and (2) Parkinson's disease: defined as a progressive disease of the nervous system characterized by muscular rigidity and tremor (*Parkinson's Disease—Symptoms and Causes*, 2020). Participants with Parkinson's disease were all studied during the “OFF” period which was defined as withdrawal of antiparkinsonian medication for 12+ h. The criteria for chronic pain in SAP was defined as the self-reported perception of a localized or generalized unpleasant bodily sensation that caused prolonged physical discomfort or mental distress. Phantom limb pain was defined as pain that is perceived as originating from an amputated limb. Movement imagery was defined as a mental execution of a movement without any muscle activation of the limb imagined to be moved that may, or may not, involve a visual cue. Movement execution task was defined as muscle activation of a limb while performing a task. Studies were excluded from analysis if: (1) stereotaxic coordinates were only reported as between-group comparisons; (2) stereotaxic coordinates included subjects with incomplete spinal cord injuries; (3) stereotaxic coordinates included Parkinson's disease subjects actively taking antiparkinsonian medications; (4) stereotaxic coordinates reported did not differentiate between the two groups of subjects with pain and subjects without pain. One reviewer used the National Institutes of Health (NIH) Quality Assessment Tool for Observational Cohort and Cross-Sectional Studies (see Supplementary Material S2) to assess each study's internal validity and assign a rating of good, fair, or poor. Stereotaxic coordinates were extracted and placed into one of the following groups primarily based on their involvement of a SAP or MAP cohort, then movement execution or movement imagery, and with or without chronic pain. Healthy controls were evaluated from each respective study. Due to lack of articles studying movement imagery or self-reported pain in the MAP cohort and limited by articles reporting of within-group stereotaxic coordinates, movement imagery and pain was solely analyzed in the SAP cohort.

### ALE/Statistical Analysis

Individual stereotaxic coordinates were extracted from respective articles and stored into an excel sheet by the first reviewer. If stereotaxic coordinates were reported as Talairach coordinates, the BioImage Suite 2.0 MNI 2 Talairach Converter web application (BioImage Suite MNI<->TAL, 2020) was used to convert to MNI space. Coordinates were then converted into txt format to be inserted into the software program, *GingerALE*. All coordinates were evaluated using the meta-analytic technique embedded in the *GingerALE* software program that evaluates the overlap between individual stereotaxic coordinates by modeling probabilistic distributions from their coordinate centers. From the generated probabilistic distributions, activation likelihood estimates are generated (see Eickhoff et al., 2009, Human Brain Mapping). A single dataset analysis *via GingerALE* was performed on each of the groups listed in Table 2. Contrast dataset analyses were performed under four connditions: (1) Altered afferent feedback during movement execution: comparing SAP cohort relative to healthy controls), (2) Altered afferent feedback in persons *with pain* during movement execution: SAP cohort with pain relative to healthy controls, (3) Altered afferent feedback during movement imagery: SAP cohort relative to healthy controls, and (4) Altered efferent motor commands during movement execution: MAP cohort relative to healthy controls. The single dataset analysis had a cluster forming threshold of p=0.005. The contrast threshold was set to *p* = 0.05 with a minimum cluster volume of 200 mm^3^. Brain regions reported in **Tables 5**–**7** used the Harvard-Oxford Cortical Structural Atlas and the Cerebellar Atlas in MNI152 space after normalization with FMRIB's (Functional Magnetic Resonance Imaging of the Brain) Non-linear Image Registration Tool (FNIRT) from the FSL (FMRIB Software Library) program (Douaud, 2016), reporting the label with the highest probability.

**Table 2:** List of groups and abbreviations for single dataset analysis.

	**Group**	**Abbreviation**
SAP	Healthy controls—movement execution	HC ME
	Healthy controls—movement imagery	HC MI
	Sensory altered processing without pain—movement execution	SAP NP ME
	Sensory altered processing without pain—movement imagery	SAP NP MI
	Sensory altered processing with pain—movement execution	SAP w/P ME
MAP	Healthy controls—movement execution	HC ME
	Motor altered processing—movement execution	MAP ME

*SAP, Sensory Altered Processing; MAP, Motor Altered Processing*.

## Results

### Database Search

**Figure 2:**
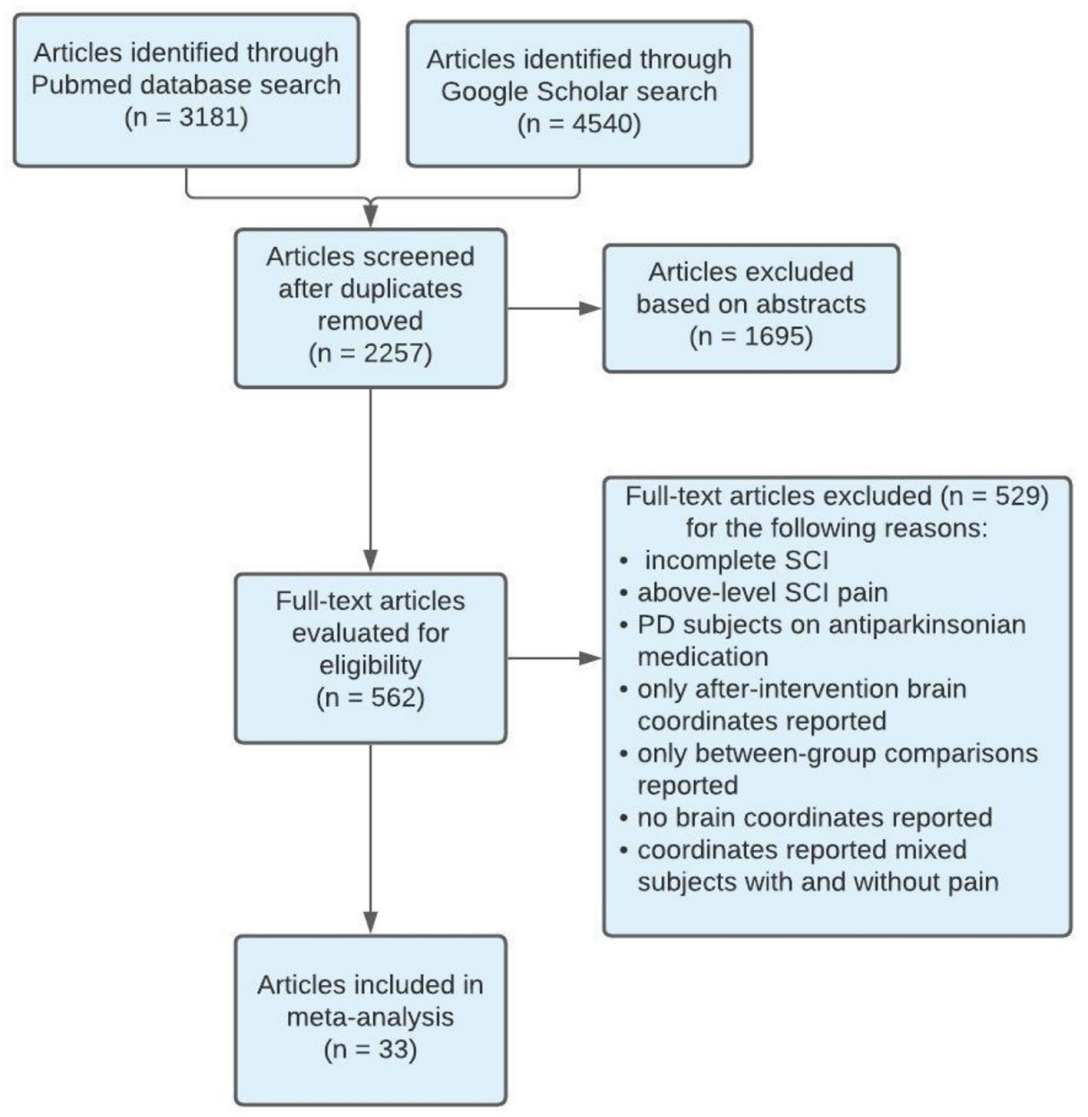
PRISMA flow diagram of search methodology for articles included in meta-analysis. SCI, Spinal Cord Injury; PD, Parkinson's disease.

Of the included studies, stereotaxic coordinates were extracted from 33 experiments, totaling 651 subjects and 968 coordinates. An example of the search methodology is included in Table 3 to demonstrate how keywords were used to find related articles. Additionally, Figure 2 displays the screening process of the database search as articles were excluded or kept for the meta- analysis.

**Table 3:** Example of keyword search methodology used to identify studies for meta-analysis.

**Search stage**	**Search terms**	**Number of hits**
1	“Spinal Cord Injuries” [Mesh]	58,950
2	“Magnetic Resonance Imaging” [Mesh]	576,654
3	Movement execution	14,101
4	Spinal cord injuries AND magnetic execution	Resonance imaging AND movement 7

Studies used for SAP cohort included SCI and amputee participants with or without chronic pain and healthy controls performing a movement execution or movement imagery task. The tasks that ranged from simple (plantar flexion) to moderate (hand movement tasks) difficulty. Imaging modalities used were either PET or fMRI. All included studies were a level II cohort study using levels of evidence pyramid by Forrest and Miller (Forrest and Miller, 2016) (see Supplementary Material S3). Some studies were rated as fair quality due to differences in the study population, lack of inclusion or exclusion criteria, or lack of statistical adjustment for confounding variables. See Table 4 for characteristics of studies for SAP.

**Table 4:** Characteristics of studies included in the meta-analysis measuring cortical activity during movement imagery and movement execution in SAP.

	**Study**	**NIH QR**	**Imaging modality**	**Participants (*n*)**	**Task performed**
SAP					
SCI	Curt et al. (2002)	Good	PET	SCI NP (7), HC (8)	Exec: R wrist extension
	Cramer et al. (2005)	Fair	fMRI	SCI NP (12), HC (12)	Exec: R plantar flexion MI: R plantar flexion
	Hotz-Boendermaker et al. (2008)	Good	fMRI	SCI NP (9), HC (12)	Exec: R dorsal and plantar flexion MI: R dorsal and plantar flexion
	Alkadhi et al. (2005)	Good	fMRI	SCI NP (8), HC (8)	Exec: R dorsal and plantar flexion MI: R dorsal and plantar flexion
Limb amputation	Roux et al. (2003)	Fair	fMRI	Amp PLP (10), HC (10)	Exec: R and L flexion and extension of fingers or toes
	Diers et al. (2010)	Good	fMRI	Amp PLP (7), Amp Non-PLP (7), HC (9)	Exec: R and L make a fist MI: R and L make a fist
	Lotze et al. (2001)	Good	fMRI	Amp PLP (7), Amp Non-PLP (7), HC (7)	Exec: R and L make a fist MI: R and L make a fist
	MacIver et al. (2008)	Good	fMRI	Amp PLP (13), HC (6)	Exec: R and L opening and closing of a fist MI: R and L opening and closing of a fist
	Raffin et al. (2012)	Fair	fMRI	Amp Non-PLP (14)	Exec: R and L opening and closing of a fist MI: R and L opening and closing of a fist
	Zheng et al. (2021)	Good	fMRI	Amp PLP (10), Amp Non-PLP (10), HC (10)	Exec: R and L movement of big toe MI: R and L movement of big toe
	Romero-Romo et al. (2010)	Fair	fMRI	Amp Non-PLP (6), HC (6)	Exec: R and L flexion and extension of toes MI: R and L flexion and extension of toes
	Duarte et al. (2020)	Good	fMRI	Amp PLP (18)	Exec: R and L dorsal and plantar flexion
	Foell et al. (2013)	Fair	fMRI	Amp PLP (11)	Exec: Lip pursing and R and L hand movement tasks
	Yu et al. (2014)	Good	fMRI	Amp Non-PLP (6)	Exec: R and L tapping toes

*NIH, National Institutes of Health; QR, Quality Rating; SAP, Sensory Altered Processing; SCI, Spinal Cord Injury; Amp, Amputee; PLP, Phantom Limb Pain; Non-PLP, Non-Phantom Limb Pain; HC, Healthy Controls; NP, No Pain; fMRI, functional Magnetic Resonance Imaging; PET, Positron Emission Tomography; Exec, Movement Execution; MI, Movement Imagery; R, right; L, left*.

Studies used for MAP included participants with Parkinson's disease and dystonia without pain and healthy controls performing a movement execution task. The tasks performed ranged from simple (finger tapping) to complex (writing). Imaging modalities used were either PET or fMRI. All included studies were a level II cohort study using levels of evidence pyramid by Forrest and Miller (Forrest and Miller, 2016) (see Supplementary Material S3). Some studies were rated as fair quality due to differences in the study population, lack of inclusion or exclusion criteria, or lack of statistical adjustment for confounding variables. See Table 5 for characteristics of studies for MAP.

**Table 5:** Characteristics of studies included in the meta-analysis measuring cortical activity during movement execution in MAP.

	**Study**	**NIH QR**	**Imaging modality**	**Participants (*n*)**	**Task performed**
MAP					
Dystonia	de Vries et al. (2008)	Good	fMRI	Dys (8), HC (9)	R wrist flexion/extension, fist clenching
	Kadota et al. (2010)	Good	fMRI	Dys (7), HC (10)	R and L hand tapping
	Preibisch et al. (2001)	Good	fMRI	Dys (12), HC (10)	R writing
	Lerner et al. (2004)	Good	PET	Dys (10), HC (10)	R hand tapping, writing
	Ibáñez et al. (1999)	Fair	PET	Dys (7), HC (7)	R hand tapping, writing, fist sustained contraction
	Ceballos-Baumann et al. (1995)	Good	PET	Dys (6), HC (6)	R hand joystick movement
	Playford et al. (1998)	Good	PET	Dys (6), HC (6)	R hand joystick movement
Parkinson's disease	Baglio et al. (2011)	Good	fMRI	PD (15), HC (11)	R finger button press
	Cerasa et al. (2006)	Good	fMRI	PD (10), HC (11)	R finger tapping
	Haslinger et al. (2001)	Good	fMRI	PD (8), HC (8)	R hand joystick movement
	Katschnig et al. (2011)	Good	fMRI	PD (20), HC (20)	R and L ankle dorsiflexion
	Kraft et al. (2009)	Good	fMRI	PD (12), HC (12)	R and L hand button press
	Maillet et al. (2012)	Good	fMRI	PD (12)	R hand joystick movement
	Mallol et al. (2007)	Good	fMRI	PD (13), HC (11)	R and L hand movements
	Sabatini et al. (2000)	Fair	fMRI	PD (6), HC (6)	R finger to thumb opposition, making and clenching fist
	Yu et al. (2007)	Good	fMRI	PD (8), HC (8)	R thumb button pressing
	Zhao et al. (2014)	Good	fMRI	PD (21), HC (22)	R finger tapping
	Yan et al. (2015)	Fair	fMRI	PD (11), HC (12)	R and L finger to thumb opposition
	Schwingenschuh et al. (2013)	Good	fMRI	PD (20), HC (10)	R and L ankle dorsiflexion

*NIH, National Institutes of Health; QR, Quality Rating; MAP, Motor Altered Processing; Dys, Dystonia; PD, Parkinson's Disease; HC, Healthy Controls; fMRI, functional Magnetic Resonance Imaging; PET, Positron Emission Tomography; R, right; L, left*.

### Meta-Analysis

See Tables 6–8 for significant results found in activated brain regions with stereotaxic coordinates in MNI space identified by the ALE meta-analyses.

**Table 6:** Activated brain regions within each between-group contrast for SAP performing a movement execution task.

	**Cluster #**	**Volume mm^**3**^**	***P*-value**	***Z*-value**	**x, y, z**	**Brain region**
Movement execution in SAP cohort						
HC > No pain	1	2,752	0.0076	2.43	49.8, 2.9, 13.4	R Precentral Gyrus
	2	2,040	0.0033	2.72	−9.6, 0, 45	L Juxtapositional Lobule Cortex (SMA)
No pain > HC	1	800	0.0041	2.64	9.4, −6, 68.8	R Juxtapositional Lobule Cortex (SMA)
	2	384	0.0052	2.56	−36, −22, 56	L Precentral Gyrus
No pain > pain	1	888	0.0094	2.35	6.8, −6.5, 70.8	R Juxtapositional Lobule Cortex (SMA)
Pain > No pain	1	936	0.0312	1.86	−28, −40, 54	L Superior Parietal Lobule

*MNI coordinates (x, y, z) of brain regions surviving a cluster threshold of p < 0.05 for contrast studies and a cluster forming threshold for p < 0.005 for single studies. SAP, Sensory Altered Processing; HC, Healthy Controls; NP, No Pain; w/ P, with Pain; L, Left; R, Right; ALE, Activation Likelihood Estimate. Brain labels automatically generated in GingerALE using the MNI space*.

**Table 7:** Activated brain regions within each between-group contrast for SAP performing a movement imagery task.

	**Cluster #**	**Volume mm^**3**^**	***P*-value**	***Z*-value**	**x, y, z**	**Brain region**
Movement imagery of SAP cohort						
HC > no pain	1	3,224	0.0024	2.82	0, 0, 60	L Juxtapositional Lobule Cortex (SMA)
No pain > HC	–	–	–	–	–	–

*MNI coordinates (x, y, z) of brain regions surviving a cluster threshold of p < 0.05 for contrast studies and a cluster forming threshold for p < 0.005 for single studies. SAP, Sensory Altered Processing; HC, Healthy Controls; NP, No Pain; L, Left; ALE, Activation Likelihood Estimate. Brain labels automatically generated in GingerALE using the MNI space*.

**Table 8:** Activated brain regions within each between-group contrast for MAP cohort performing a movement execution task.

	**Cluster #**	**Volume mm^**3**^**	***P*-value**	***Z*-value**	**x, y, z**	**Brain region**
HC > MAP Cohort						
	1	1,552	0.0018	2.91	−12, 2, 60	L Superior Frontal Gyrus
	2	1,360	0.003	2.75	−20, −22, −4	L Thalamus
	3	816	0.0058	2.52	−42, −4, 8	L Insular Cortex
	4	560	0.0045	2.61	58, −36, 22	R Planum Temporale, R Supramarginal Gyrus,
						posterior division
	5	312	0.0193	2.07	−42, −36, 60	L Postcentral Gyrus
MAP cohort > HC	1	608	0.0273	1.92	30, −68, −32	R Cerebellum Crus I
	2	448	0.0234	1.99	39, −46, 41	R Supramarginal Gyrus,
						posterior division
	3	360	0.0039	2.66	30, −18, 50	R Precentral Gyrus
	4	208	0.0231	1.99	−8, −50, −26	L Cerebellum I–IV

*MNI coordinates (x, y, z) of brain regions surviving a cluster threshold of p < 0.05 for contrast studies and a cluster forming threshold for p < 0.005 for single studies. MAP, Motor Altered Processing; HC, Healthy Controls; L, Left, R, Right; ALE, Activation Likelihood Estimate. Brain labels automatically generated in GingerALE using the MNI space*.

### SAP Cohort Analysis Results

**Figure 3:**
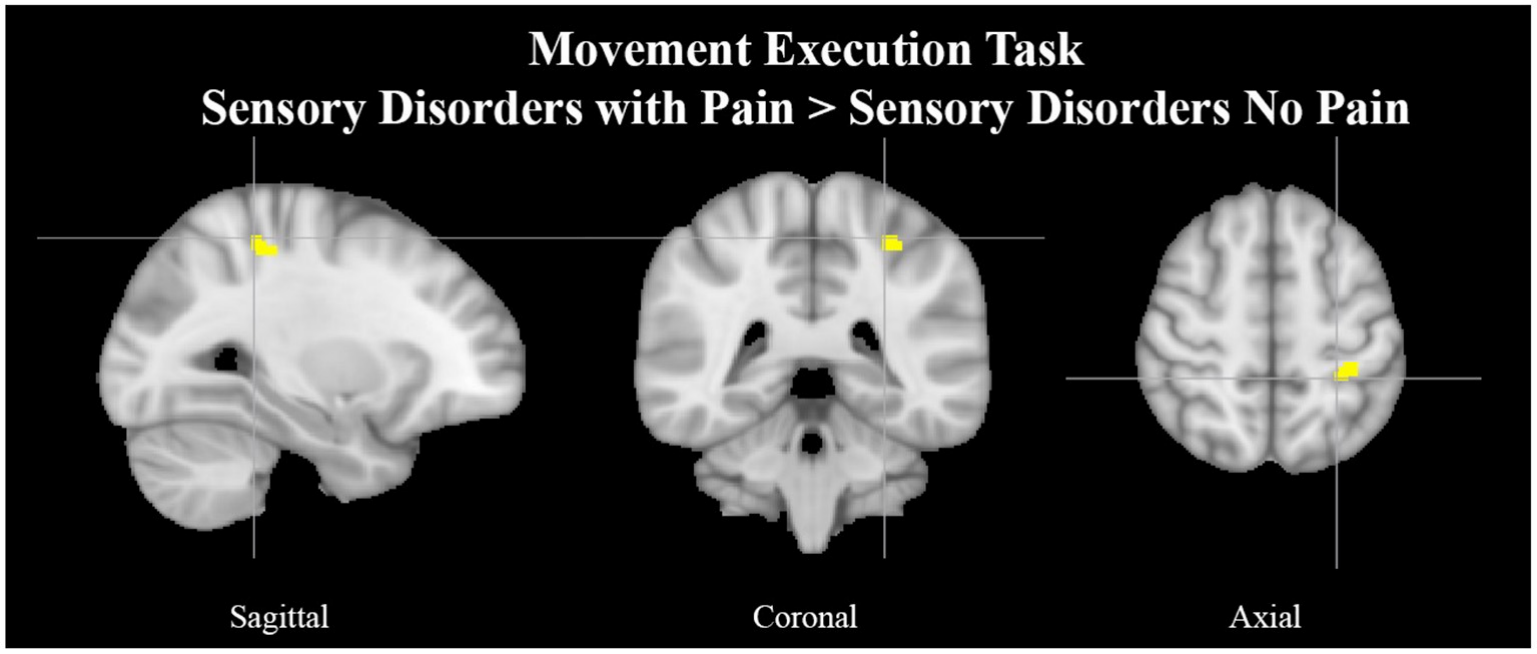
Left superior parietal lobule activation in SAP cohort with pain. Exemplar of a between-group activation peak of the movement execution contrast SAP w/P > SAP NP. Yellow-colored regions show increased brain activity in the left superior parietal lobule (cross- hairs reflect peak activation peak *x* = −28, *y* = −40, *z* = 54). SAP, Sensory Altered Processing; w/P, with Pain; NP, No Pain.

See Tables 6, 7 for more detailed information of activation clusters. A peak activation was found in the right precentral gyrus and the left juxtapositional lobule cortex for the movement execution contrast HC > SAP NP. A peak activation was found in the right juxtapositional lobule cortex and the left precentral gyrus for the movement execution contrast SAP NP > HC. One activation peak was found in the right juxtapositional lobule cortex for the movement execution contrast SAP NP > SAP w/P. One activation peak was found in the left superior parietal lobule for the movement execution contrast SAP w/P > SAP NP (see Figure 3). One activation peak was found in the left juxtapositional lobule cortex for the movement imagery contrast HC > SAP NP. No activations were found in the movement imagery contrast SAP NP > HC.

### MAP Cohort Analysis Results

**Figure 4:**
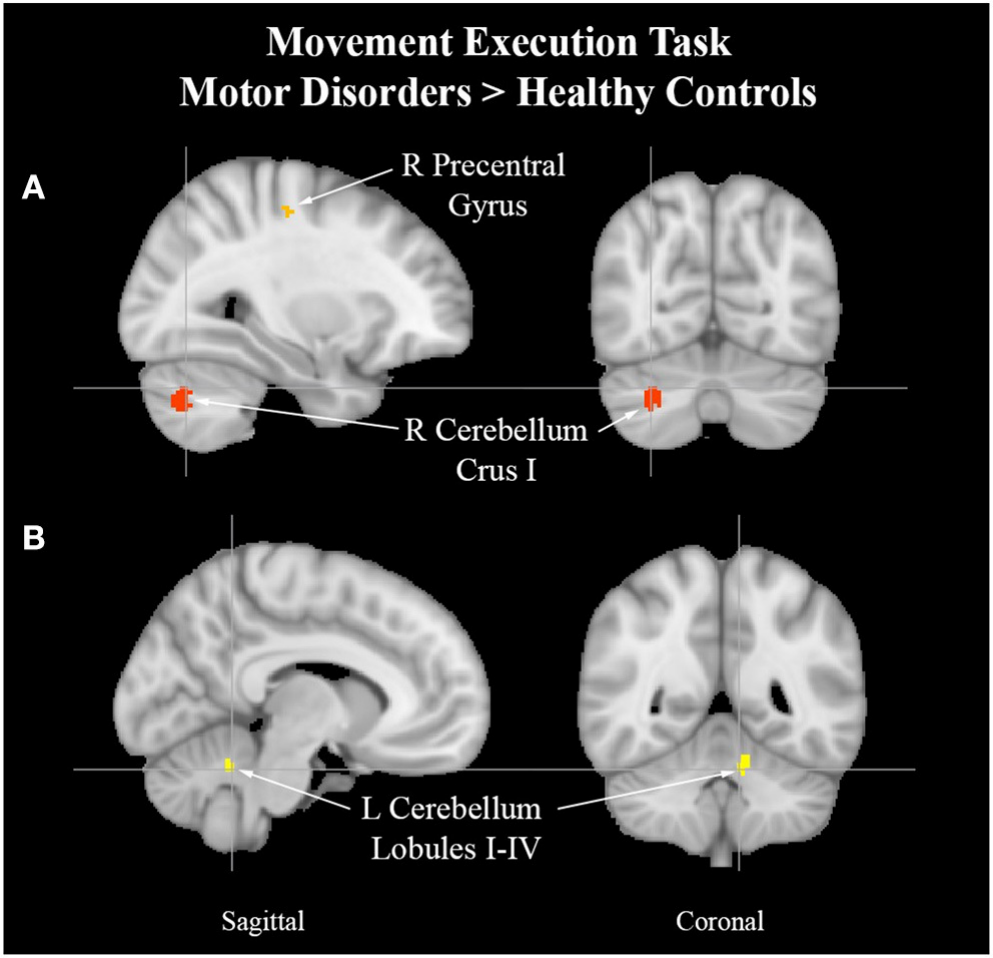
Cerebellum activation peaks in MAP cohort. Exemplar of between-group activation peaks of the movement execution contrast MAP > HC. A. Orange-colored region shows increased brain activity in the right precentral gyrus. Red-colored regions show increased brain activity in the right cerebellum Crus I (cross-hairs reflect activation peak *x* = 30, y = −68, *z* = −32). B. Yellow-colored regions show increased brain activity in the left cerebellum lobules I–IV (cross-hairs reflect activation peak *x* = −8, *y* = −50, *z* = −26).

See Table 8 for more detailed information of activation clusters. Five activation peaks were found in the movement execution contrast HC > MAP. Four activation peaks were found in the movement execution contrast MAP > HC (see Figure 4).

## Discussion

Brain activity in persons with atypical afferent input from peripheral nerve or spinal cord injury is modulated based on the presence of pain. Sensorimotor integration is tightly coupled in the central nervous system and depends on feedback from the peripheral nervous system for proprioceptive input. The primary motor cortex is extensively interconnected with descending pain modulatory regions and sensory processing areas of the brain (Holmes et al., 2021). It remains unclear how the presence of pain modulates central nervous system activity in persons with spinal cord injuries or limb loss. In this ALE meta-analysis, we show (1) that the supplementary motor area has greater activity in persons with atypical afferent activity in persons with *no pain* vs. *in pain*, and (2) that above-normal efferent motor activity is associated with decreased activity in brain regions involved in affective processing. Findings are outlined in the context of sensorimotor integration.

### Changes in Afferent Input and the Perception of Pain

Motor regions are active in persons who do not report elevated pain. The SMA is a brain region located anterior to M1 and is involved in motor planning and learning and prediction (Makoshi et al., 2011). We offer two suggestions for observed differences in brain activity (1) an attempt to engage descending pain modulatory regions of the brain that are downstream from motor-regions or (2) the motor system updating itself to the absence of limb proprioceptive input to sustain behavioral goals and rectify discrepant motor programs.

Engagement of motor regions within the brain, either through endogenous or exogenous sources, have shown to be effective sources of analgesia (Holmes et al., 2021). The use of repetitive transcranial magnetic stimulation or physical therapy-based programs have shown possible to decrease pain symptoms through motor-based approaches (Gatzinsky et al., 2021). At a network level, engagement of the motor system (e.g., M1) can produce downstream effects on descending pain modulatory regions (e.g., periaqueductal gray) that may act through the brain stem, locus coeruleus, nucleus raphe magnus, and nucleus reticularis gigantocellularis to decrease pain symptoms (Holmes et al., 2021). Findings from the current investigation contrast what would be predicted from this hypothesis as no sub-cortical regions were reported when comparing persons with atypical afferent input with and without pain. Notably, this may be due to the meta-analytic nature of this investigation and loss of statistical resolution. Follow-up investigations that include online neuroimaging to differentiate nociceptive input from pain symptom reporting will be required to fully test this hypothesis.

Atypical afferent processing of nociceptive stimuli may drive motor planning. Comparing cohorts with altered afferent input based on the presence or absence of pain suggests a role for peripherally derived updating of motor programs. That is, M1 has immense connectivity with S1 through corticocortical connectivity, direct connectivity, sub-cortical relays and through peripheral engagement of motor units and sensory neurons (Umeda et al., 2019). The absence, or diminished capacity, of the motor system in persons with a physical disability due to limb loss or spinal cord injury may trigger the SMA to engage novel motor programs that sustain, or attempt to sustain, task goals (Makoshi et al., 2011). Alternatively, having been recently recognized to have a role in the attentional modulation of pain, a continued increased activity within the left superior parietal lobule (Makoshi et al., 2011) could suggest a “voluntary” amplification of attention toward stimuli during movement. While suffering from chronic pain conditions, increased attention to pain (McCracken, 1997; Bushnell et al., 2013) during movement could lead to a conditioned expectation of pain from a motor execution task that produces no noxious stimuli. In other words, the anticipation of pain (Poppe et al., 2011; Zeidan et al., 2015) becomes a learned behavior in which any motor activity can act as a pain-predictive cue and subsequent movements can drive motor relearning in the context of pain (Dancey et al., 2016). Increased activity with the SMA would suggest engagement of motor planning structures that could be used to tune motor control in line with sensory feedback. This highlights the potential of physical exercise interventions to target both the physical and cognitive experience of pain in programs designed to stress rehabilitative sensorimotor integration.

### Changes in Efferent Output and Pain Connectome Suppression

Changes observed in the cohort with atypical efferent motor control may offer insight into the role of the motor system in pain processing. Uncoupling of S1-M1 functional communication has been observed in hyper-kinetic movement disorders such as dystonia (Melgari et al., 2013). Along the lines of a centrally mediated source for elevated motor output, the ALE analysis revealed increased activity in the right precentral gyrus which is a region implicated in the initiation and control of voluntary movement (Papale and Hooks, 2018). Two ALE clusters of the left cerebellum lobules I-IV and the right cerebellum Crus I in subjects with MAP suggests attempts at rectifying incongruence between sensory and motor processes (Stoodley et al., 2012; Mehnert and May, 2019) and online adjustments to motor output.

Decreased activity was found in persons with atypical efferent processing relative to healthy controls in regions implicated in the pain connectome (Kucyi and Davis, 2015; Coghill, 2020). Most salient differences were found in left thalamus and the left postcentral gyrus which have roles in the primary interception of afferent sensory input to the brain (Yen and Lu, 2013). Decreased activity was also observed in the left insular cortex, a region that links sensory experience with emotional value (Nieuwenhuys, 2012) and has been shown to have increased activity in persons with chronic pain (Starr et al., 2009). Observations of decreased activity within the left superior frontal gyrus, a region involved in working memory that influences decision making and goal-driven behavior (du Boisgueheneuc et al., 2006) may provide insight to the nature of the hyper-kinetic movement disorders included, placing emphasis on lower order central nervous system regions such as M1 and sub-cortical structures including the basal ganglia (Mink, 2003). Alternatively, observed depression of activity within frontal structures may underscore a relative ease of engaging motor processes in persons with sustained output, as a form of motor practice (Wright et al., 2012). Observing mirrored activation of the precentral gyrus and juxtapositional lobule cortex in opposite hemispheres amongst the SAP with no pain cohort and healthy controls may reflect inter-hemispheric functional compensatory adaptation. In particular, such patterns of activation have been observed in cases of stroke where the intact contralateral representation of the ipsilateral lesion may show greater relative activation in response to the damaged area to preserve behavior performance (Takatsuru et al., 2013). More recent data suggests that this process of contralateral may be inflammatory-mediated as inflammation has been observed in regions contralateral to a primary lesion site (Lucas-Ruiz et al., 2021), perhaps giving insight into modulating the local synaptic environment and the suppression of motor system activity in the SAP cohort experiencing chronic pain. We suggest that the alterations observed in the sensorimotor system likely interfere with the distributed nociceptive system which could impact the way that nociceptive stimuli are processed into pain symptom reporting.

### Integrating Perspectives

**Figure 5:**
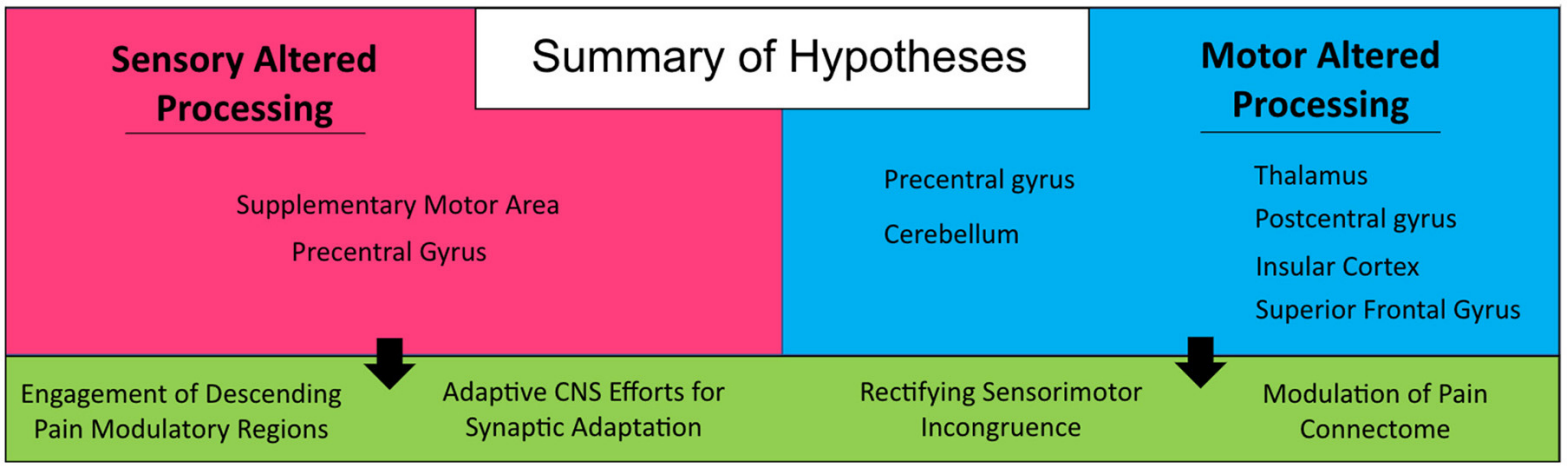
Summary of hypotheses. Generated from findings in the current investigation on persons with sensory and motor altered processing.

The motor system has an important role in pain management. Acute pain, which comes on quickly and lasts for a short time (<3 months) as opposed to the long-lasting nature of chronic pain (>3 months), has been shown to have an inhibitory effect on the motor cortex (Boudreau et al., 2007; Mercier and Léonard, 2011). However, the relationship of chronic pain and motor cortex reorganization is more complex and unclear whether one causes the other. Chronic pain, either in terms of prolonged nociceptive input or through central sensitization, can have a debilitating impact on human motor behavior that is often neglected in pain treatment services. This negative motor-effect can have malignant tendencies, translating into social isolation, devolving mental health, and potential life-threatening health conditions. The focus of this investigation was to provide hypothesis generating analyses pertaining to the relative impact on the brain of atypical efferent and afferent processing of motor and sensory information, respectively. Findings provide support for four hypotheses between the two cohorts evaluated in this investigation (see Figure 5) and provide insight into the impact of motor processes on central brain regions implicated in nociceptive processing. First, increased engagement of the supplementary motor area in persons in the SAP cohort suggests a prominent role for the motor system in responding to pain, either in terms of engaging pain modulatory regions (Hypothesis 1) or in terms of engaging adaptive motor programs in response to elevated nociceptive signaling, minimizing the use of motor programs that result in pain (Hypothesis 2). Alternatively, in the MAP cohort, there is evidence to suggest again attempts at adaptation, but focused on incongruence between deficient motor programs and sensory expectations (Hypothesis 3) and the intended suppression of brain regions (e.g., Thalamus) implicated in pain processing (Hypothesis 4). Notably, these hypotheses are not mutually exclusive, and are conceptually mirrored between cohorts (focused on adaptation and pain modulation). Repetitive occurrence of pain can form a type of muscle memory within the nociceptive system, perhaps resulting from failures at engaging adaptive circuitry or pain modulatory regions that leaves individuals more susceptive to the development of chronic pain conditions (Garcia-Larrea and Bastuji, 2018; McCarberg and Peppin, 2019). As physical therapy regimens have proved to be helpful in the treatment of both sensory and motor disorders (Allen et al., 2015; Borisovskaya et al., 2020; Zaheer et al., 2021), we suggest the motor system may also play an important role in chronic pain management, in terms of both adaptive motor programs and pain modulatory efforts.

## Limitations

There are limitations that require mention based on the ALE meta-analytic approach adopted: (1) included cohorts: atypical afferent and efferent processing represents the basis for a large myriad of neurological conditions. Including other conditions such as multiple sclerosis or amyotrophic lateral sclerosis may impact the specificity of current findings; (2) hemispheric laterality: based on the limited sample of articles and bilateral projections of sensory and motor circuits, we elected not to split our data based on affected limb; (3) reporting bias: based on the secondary data analysis approach we are limited to the reported data of each study. However, these may be limited to within-group, rather than between-group, stereotaxic coordinates, no reporting of pain, differential reporting basis of pain symptoms, and different clinical standards for neurological disorders.

## Conclusion

Central nervous system processing of nociceptive input is the basis for understanding acute and chronic pain. To date, research and clinical interests has focused on the primary reception and distributed nature of nociception on the brain; however, there is growing interest on the impact of efferent motor processes on sensory processing. Looking on the other side of sensory processing offers a non-pharmacological source of analgesia that has transcending impacts on physical and mental health. Findings from this investigation point toward an important role of efferent motor processes on dampening central processing of nociception and motor learning in the context of pain.

## Data Availability Statement

The original contributions presented in the study are included in the article/Supplementary Material, further inquiries can be directed to the corresponding author/s.

## Author Contributions

Study conception and design, analysis and interpretation of results, and draft manuscript preparation: CG and SH. Data collection: CG. Both authors reviewed the results and approved the final version of the manuscript.

## Funding

This work was supported by the Department of Anesthesia, Critical Care and Pain Medicine, Boston Children's Hospital, Boston MA (Awarded to SH).

## Conflict of Interest

The authors declare that the research was conducted in the absence of any commercial or financial relationships that could be construed as a potential conflict of interest.

## Publisher's Note

All claims expressed in this article are solely those of the authors and do not necessarily represent those of their affiliated organizations, or those of the publisher, the editors and the reviewers. Any product that may be evaluated in this article, or claim that may be made by its manufacturer, is not guaranteed or endorsed by the publisher.

## References

[B1] Alkadhi H. Brugger P. Boendermaker S. H. Crelier G. Curt A. Hepp-Reymond M.-C. . (2005). What disconnection tells about motor imagery: Evidence from paraplegic patients. Cereb. Cortex. 15, 131–140. 10.1093/cercor/bhh116 15238440

[B2] Allen N. E. Moloney N. van Vliet V. Canning C. G. (2015). The rationale for exercise in the management of pain in Parkinson's disease. J. Parkinsons Dis. 5, 229–239. 10.3233/JPD-140508 25649828 PMC4923748

[B3] Ambrose K. R. Golightly Y. M. (2015). Physical exercise as non-pharmacological treatment of chronic pain: why and when. Best Pract. Res. Clin. Rheumatol. 29, 120–130. 10.1016/j.berh.2015.04.022 26267006 PMC4534717

[B4] Angel R. W. Malenka R. C. (1982). Velocity-dependent suppression of cutaneous sensitivity during movement. Exp. Neurol. 77, 266–274. 10.1016/0014-4886(82)90244-8 7095061

[B5] Baglio F. Blasi V. Falini A. Farina E. Mantovani F. Olivotto F. . (2011). Functional brain changes in early Parkinson's disease during motor response and motor inhibition. Neurobiol. Aging. 32, 115–124. 10.1016/j.neurobiolaging.2008.12.009 19188005

[B6] BioImage Suite MNI<->TAL (2020). Available online at: https://bioimagesuiteweb.github.io/webapp/mni2tal.html (accessed August 25, 2020).

[B7] Booth J. Moseley G. L. Schiltenwolf M. Cashin A. Davies M. Hübscher M. (2017). Exercise for chronic musculoskeletal pain: a biopsychosocial approach. Musculoskel. Care 15, 413–421. 10.1002/msc.1191 28371175

[B8] Borisovskaya A. Chmelik E. Karnik A. (2020). “Exercise and chronic pain,” in Physical Exercise for Human Health, ed J. Xiao (Singapore: Springer), 233–253. 10.1007/978-981-15-1792-1_16 32342462

[B9] Boudreau S. Romaniello A. Wang K. Svensson P. Sessle B. J. Arendt-Nielsen L. (2007). The effects of intra-oral pain on motor cortex neuroplasticity associated with short-term novel tongue-protrusion training in humans. Pain 132, 169–178. 10.1016/j.pain.2007.07.019 17870237

[B10] Buckingham G. Carey D. P. Colino F. L. deGrosbois J. Binsted G. (2010). Gating of vibrotactile detection during visually guided bimanual reaches. Exp. Brain Res. 201, 411–419. 10.1007/s00221-009-2050-8 19851758

[B11] Bushnell M. C. Ceko M. Low L. A. (2013). Cognitive and emotional control of pain and its disruption in chronic pain. Nat. Rev. Neurosci. 14, 502–511. 10.1038/nrn3516 23719569 PMC4465351

[B12] Ceballos-Baumann A. O. Passingham R. E. Warner T. Playford E. D. Marsden C. D. Brooks D. J. (1995). Overactive prefrontal and underactive motor cortical areas in idiopathic dystonia. Ann. Neurol. 37, 363–372. 10.1002/ana.410370313 7695236

[B13] Cerasa A. Hagberg G. E. Peppe A. Bianciardi M. Gioia M. C. Costa A. . (2006). Functional changes in the activity of cerebellum and frontostriatal regions during externally and internally timed movement in Parkinson's disease. Brain Res. Bull. 71, 259–269. 10.1016/j.brainresbull.2006.09.014 17113955

[B14] Coghill R. C. (2020). The distributed nociceptive system: a framework for understanding pain. Trends Neurosci. 43, 780–794. 10.1016/j.tins.2020.07.004 32800534 PMC7530033

[B15] Cramer S. C. Lastra L. Lacourse M. G. Cohen M. J. (2005). Brain motor system function after chronic, complete spinal cord injury. Brain J. Neurol. 128, 2941–2950. 10.1093/brain/awh648 16246866

[B16] Curt A. Alkadhi H. Crelier G. R. Boendermaker S. H. Hepp-Reymond M.-C. Kollias S. S. (2002). Changes of non-affected upper limb cortical representation in paraplegic patients as assessed by fMRI. Brain J. Neurol. 125, 2567–2578. 10.1093/brain/awf250 12390981

[B17] Daenen L. Varkey E. Kellmann M. Nijs J. (2015). Exercise, not to exercise, or how to exercise in patients with chronic pain? Applying science to practice. Clin. J. Pain 31, 108–114. 10.1097/AJP.0000000000000099 24662498

[B18] Dahlhamer J. (2018). Prevalence of chronic pain and high-impact chronic pain among adults—United States, 2016. MMWR Morb. Mortal. Wkly. Rep. 67, 1001–1006. 10.15585/mmwr.mm6736a2 30212442 PMC6146950

[B19] Dancey E. Murphy B. Andrew D. Yielder P. (2016). Interactive effect of acute pain and motor learning acquisition on sensorimotor integration and motor learning outcomes. J. Neurophysiol. 116, 2210–2220. 10.1152/jn.00337.2016 27535371 PMC5102313

[B20] de Vries P. M. Johnson K. A. de Jong B. M. Gieteling E. W. Bohning D. E. George M. S. . (2008). Changed patterns of cerebral activation related to clinically normal hand movement in cervical dystonia. Clin. Neurol. Neurosurg. 110, 120–128. 10.1016/j.clineuro.2007.09.020 18006221

[B21] Diers M. Christmann C. Koeppe C. Ruf M. Flor H. (2010). Mirrored, imagined and executed movements differentially activate sensorimotor cortex in amputees with and without phantom limb pain. Pain. 149, 296–304. 10.1016/j.pain.2010.02.020 20359825

[B22] Douaud G. (2016). FSLVBM. Oxford. Available online at: https://fsl.fmrib.ox.ac.uk/fsl/fslwiki/FSLVBM (accessed August 25, 2020).

[B23] du Boisgueheneuc F. Levy R. Volle E. Seassau M. Duffau H. Kinkingnehun S. . (2006). Functions of the left superior frontal gyrus in humans: a lesion study. Brain J. Neurol. 129(Pt 12), 3315–3328. 10.1093/brain/awl244 16984899

[B24] Duarte D. Bauer C. C. C. Pinto C. B. Saleh Velez F. G. Estudillo-Guerra M. A. Pacheco-Barrios K. . (2020). Cortical plasticity in phantom limb pain: A fMRI study on the neural correlates of behavioral clinical manifestations. Psychiatry Res. Neuroimag. 304:111151. 10.1016/j.pscychresns.2020.111151 32738724 PMC9394643

[B25] Dudgeon B. J. Gerrard B. C. Jensen M. P. Rhodes L. A. Tyler E. J. (2002). Physical disability and the experience of chronic pain. Arch. Phys. Med. Rehabil. 83, 229–235. 10.1053/apmr.2002.28009 11833027

[B26] Eickhoff S. B. Laird A. R. Grefkes C. Wang L. E. Zilles K. Fox P. T. (2009). Coordinate-based activation likelihood estimation meta-analysis of neuroimaging data: a random-effects approach based on empirical estimates of spatial uncertainty. Hum. Brain Mapp. 30, 2907–2926. 10.1002/hbm.20718 19172646 PMC2872071

[B27] Foell J. Bekrater-Bodmann R. McCabe C. S. Flor H. (2013). Sensorimotor incongruence and body perception: an experimental investigation. Front. Hum. Neurosci. 7:310. 10.3389/fnhum.2013.00310 23805095 PMC3690352

[B28] Forrest J. L. Miller S. A. (2016). *EBDM in* action: Developing competence in EB practice.

[B29] Fraser L. E. Fiehler K. (2018). Predicted reach consequences drive time course of tactile suppression. Behav. Brain Res. 350, 54–64. 10.1016/j.bbr.2018.05.010 29768186

[B30] Garcia-Larrea L. Bastuji H. (2018). Pain and consciousness. Progr. Neuro Psychopharmacol. Biol. Psychiatry 87, 193–199. 10.1016/j.pnpbp.2017.10.007 29031510

[B31] Gatzinsky K. Bergh C. Liljegren A. Silander H. Samuelsson J. Svanberg T. . (2021). Repetitive transcranial magnetic stimulation of the primary motor cortex in management of chronic neuropathic pain: a systematic review. Scand. J. Pain 21, 8–21. 10.1515/sjpain-2020-0054 32892189

[B32] Haslinger B. Erhard P. Kämpfe N. Boecker H. Rummeny E. Schwaiger M. . (2001). Event-related functional magnetic resonance imaging in Parkinson's disease before and after levodopa. Brain J. Neurol. 124, 558–570. 10.1093/brain/124.3.558 11222456

[B33] Hautasaari P. McLellan S. Koskio M. Pesonen H. Tarkka I. M. (2020). Acute exercise modulates pain-induced response on sensorimotor cortex ~20 Hz oscillation. Neuroscience 429, 46–55. 10.1016/j.neuroscience.2019.12.044 31935493

[B34] Holmes S. A. Kim A. Borsook D. (2021). The brain and behavioral correlates of motor-related analgesia (MRA). Neurobiol. Dis. 148, 105158. 10.1016/j.nbd.2020.105158 33157210

[B35] Hotz-Boendermaker S. Funk M. Summers P. Brugger P. Hepp-Reymond M.-C. Curt A. . (2008). Preservation of motor programs in paraplegics as demonstrated by attempted and imagined foot movements. NeuroImage. 39, 383–394. 10.1016/j.neuroimage.2007.07.065 17919932

[B36] Ibáñez V. Sadato N. Karp B. Deiber M. P. Hallett M. (1999). Deficient activation of the motor cortical network in patients with writer's cramp. Neurology. 53, 96–105. 10.1212/wnl.53.1.96 10408543

[B37] Jiang W. Lamarre Y. Chapman C. E. (1990). Modulation of cutaneous cortical evoked potentials during isometric and isotonic contractions in the monkey. Brain Res. 536, 69–78. 10.1016/0006-8993(90)90010-9 2085763

[B38] Juravle G. Binsted G. Spence C. (2017). Tactile suppression in goal-directed movement. Psychon. Bull. Rev. 24, 1060–1076. 10.3758/s13423-016-1203-6 27896632

[B39] Kadota H. Nakajima Y. Miyazaki M. Sekiguchi H. Kohno Y. Amako M. . (2010). An fMRI study of musicians with focal dystonia during tapping tasks. J. Neurol. 257, 1092–1098. 10.1007/s00415-010-5468-9 20143109

[B40] Katschnig P. Schwingenschuh P. Jehna M. Svehlík M. Petrovic K. Ropele S. . (2011). Altered functional organization of the motor system related to ankle movements in Parkinson's disease: Insights from functional MRI. J. Neur. Trans. 118, 783–793. 10.1007/s00702-011-0621-x 21437717

[B41] Kraft E. Loichinger W. Diepers M. Lule D. Schwarz J. Ludolph A. C. . (2009). Levodopa-induced striatal activation in Parkinson's disease: A functional MRI study. Parkinsonism Relat. Disord. 15, 558–563. 10.1016/j.parkreldis.2009.02.005 19467909

[B42] Kucyi A. Davis K. D. (2015). The Dynamic Pain Connectome- ClinicalKey. Toronto, ON. Available online at: https://www.clinicalkey.com/#!/content/playContent/1-s2.0-S0166223614002173?returnurl=nullandreferrer=null (accessed August 25, 2020).

[B43] Larsen D. B. Graven-Nielsen T. Boudreau S. A. (2019). Pain-induced reduction in corticomotor excitability is counteracted by combined action-observation and motor imagery. J. Pain 20, 1307–1316. 10.1016/j.jpain.2019.05.001 31077798

[B44] Le Pera D. Graven-Nielsen T. Valeriani M. Oliviero A. Di Lazzaro V. Tonali P. A. . (2001). Inhibition of motor system excitability at cortical and spinal level by tonic muscle pain. Clin. Neurophysiol. 112, 1633–1641. 10.1016/S1388-2457(01)00631-9 11514246

[B45] Lerner A. Shill H. Hanakawa T. Bushara K. Goldfine A. Hallett M. (2004). Regional cerebral blood flow correlates of the severity of writer's cramp symptoms. NeuroImage. 21, 904–913. 10.1016/j.neuroimage.2003.10.019 15006657

[B46] Lotze M. Flor H. Grodd W. Larbig W. Birbaumer N. (2001). Phantom movements and pain. An fMRI study in upper limb amputees. Brain J. Neurol. 124, 2268–2277. 10.1093/brain/124.11.2268 11673327

[B47] Lucas-Ruiz F. Galindo-Romero C. Albaladejo-García V. Vidal-Sanz M. Agudo-Barriuso M. (2021). Mechanisms implicated in the contralateral effect in the central nervous system after unilateral injury: focus on the visual system. Neural Regen. Res. 16, 2125–2131. 10.4103/1673-5374.310670 33818483 PMC8354113

[B48] MacIver K. Lloyd D. M. Kelly S. Roberts N. Nurmikko T. (2008). Phantom limb pain, cortical reorganization and the therapeutic effect of mental imagery. Brain J. Neurol. 131, 2181–2191. 10.1093/brain/awn124 18567624 PMC2494616

[B49] Maillet A. Krainik A. Debû B. Troprés I. Lagrange C. Thobois S. . (2012). Levodopa effects on hand and speech movements in patients with Parkinson's disease: A FMRI study. PLoS ONE. 7:e46541. 10.1371/journal.pone.0046541 23056337 PMC3467207

[B50] Makoshi Z. Kroliczak G. van Donkelaar P. (2011). Human supplementary motor area contribution to predictive motor planning. J. Mot. Behav. 43, 303–309. 10.1080/00222895.2011.584085 21732868

[B51] Mallol R. Barrós-Loscertales A. López M. Belloch V. Parcet M. A. Avila C. (2007). Compensatory cortical mechanisms in Parkinson's disease evidenced with fMRI during the performance of pre-learned sequential movements. Brain Res. 1147, 265–271. 10.1016/j.brainres.2007.02.046 17368575

[B52] McCarberg B. Peppin J. (2019). Pain pathways and nervous system plasticity: learning and memory in pain. Pain Med. 20, 2421–2437. 10.1093/pm/pnz017 30865778

[B53] McCracken L. M. (1997). “Attention” to pain in persons with chronic pain: a behavioral approach. Behav. Ther. 28, 271–284. 10.1016/S0005-7894(97)80047-0

[B54] Mehnert J. May A. (2019). Functional and structural alterations in the migraine cerebellum. J. Cereb. Blood Flow Metab. 39, 730–739. 10.1177/0271678X17722109 28737061 PMC6446424

[B55] Melgari J. M. Zappasodi F. Porcaro C. Tomasevic L. Cassetta E. Rossini P. M. . (2013). Movement-induced uncoupling of primary sensory and motor areas in focal task-specific hand dystonia. Neuroscience 250, 434–445. 10.1016/j.neuroscience.2013.07.027 23876327

[B56] Mercier C. Léonard G. (2011). Interactions between pain and the motor cortex: insights from research on phantom limb pain and complex regional pain syndrome. Physiother. Can. 63, 305–314. 10.3138/ptc.2010-08p 22654236 PMC3157990

[B57] Mink J. W. (2003). The Basal Ganglia and involuntary movements: impaired inhibition of competing motor patterns. Arch. Neurol. 60, 1365–1368. 10.1001/archneur.60.10.1365 14568805

[B58] Nieuwenhuys R. (2012). The Insular Cortex, Vol. 195. Elsevier Science and Technology, 123–163. 10.1016/B978-0-444-53860-4.00007-6 22230626

[B59] Page M. J. McKenzie J. E. Bossuyt P. M. Boutron I. Hoffmann T. C. Mulrow C. D. . (2021). The PRISMA 2020 statement: an updated guideline for reporting systematic reviews. BMJ 372, n71. 10.1136/bmj.n71 33782057 PMC8005924

[B60] Papale A. E. Hooks B. M. (2018). Circuit changes in motor cortex during motor skill learning. Neuroscience 368, 283–297. 10.1016/j.neuroscience.2017.09.010 28918262 PMC5762136

[B61] Playford E. D. Passingham R. E. Marsden C. D. Brooks D. J. (1998). Increased activation of frontal areas during arm movement in idiopathic torsion dystonia. J. Mov. Disord. Soc. 13, 309–318. 10.1002/mds.870130218 9539346

[B62] Poppe C. Crombez G. Devulder J. Hanoulle I. Vogelaers D. Petrovic M. (2011). Personality traits in chronic pain patients are associated with low acceptance and catastrophizing about pain. Acta Clin. Belg. 66, 209–215. 10.2143/ACB.66.3.2062549 21837930

[B63] Preibisch C. Berg D. Hofmann E. Solymosi L. Naumann M. (2001). Cerebral activation patterns in patients with writer's cramp: A functional magnetic resonance imaging study. J. Neurol. 248, 10–17. 10.1007/s004150170263 11266013

[B64] Raffin E. Mattout J. Reilly K. T. Giraux P. (2012). Disentangling motor execution from motor imagery with the phantom limb. Brain J. Neurol. 135, 582–595. 10.1093/brain/awr337 22345089

[B65] Romero-Romo J. I. Bauer C. C. C. Pasaye E. H. Gutiérrez R. A. Favila R. Barrios F. A. (2010). Abnormal functioning of the thalamocortical system underlies the conscious awareness of the phantom limb phenomenon. Neuroradiol J. 23, 671–679. 10.1177/197140091002300605 24148720

[B66] Roux F.-E. Lotterie J.-A. Cassol E. Lazorthes Y. Sol J.-C. Berry I. (2003). Cortical areas involved in virtual movement of phantom limbs: Comparison with normal subjects. Neurosurgery. 53, 1342–1352. 10.1227/01.neu.0000093424.71086.8f 14633300

[B67] Sabatini U. Boulanouar K. Fabre N. Martin F. Carel C. Colonnese C. . (2000). Cortical motor reorganization in akinetic patients with Parkinson's disease: A functional MRI study. Brain J. Neurol. 123, 394–403. 10.1093/brain/123.2.394 10648446

[B68] Schwingenschuh P. Katschnig P. Jehna M. Koegl-Wallner M. Seiler S. Wenzel K. . (2013). Levodopa changes brain motor network function during ankle movements in Parkinson's disease. J. Neural. Trans. 120, 423–433. 10.1007/s00702-012-0896-6 22990677

[B69] Seki K. Fetz E. E. (2012). Gating of sensory input at spinal and cortical levels during preparation and execution of voluntary movement. J. Neurosci. 32, 890–902. 10.1523/JNEUROSCI.4958-11.2012 22262887 PMC3293372

[B70] Starr A. Cohen L. G. (1985). ‘Gating' of somatosensory evoked potentials begins before the onset of voluntary movement in man. Brain Res. 348, 183–186. 10.1016/0006-8993(85)90377-4 4063823

[B71] Starr C. J. Sawaki L. Wittenberg G. F. Burdette J. H. Oshiro Y. Quevedo A. S. . (2009). Roles of the insular cortex in the modulation of pain: insights from brain lesions. J. Neurosci. 29, 2684–2694. 10.1523/JNEUROSCI.5173-08.2009 19261863 PMC2748680

[B72] Stoodley C. J. Valera E. M. Schmahmann J. D. (2012). Functional topography of the cerebellum for motor and cognitive tasks: an fMRI study. Neuroimage 59, 1560–1570. 10.1016/j.neuroimage.2011.08.065 21907811 PMC3230671

[B73] Takatsuru Y. Nakamura K. Nabekura J. (2013). Compensatory contribution of the contralateral pyramidal tract after experimental cerebral ischemia. Clin. Recov. CNS Damage 32, 36–44. 10.1159/000346409 23859961

[B74] Umeda T. Isa T. Nishimura Y. (2019). The somatosensory cortex receives information about motor output. Sci. Adv. 5, eaaw5388. 10.1126/sciadv.aaw5388 31309153 PMC6620090

[B75] Voudouris D. Broda M. D. Fiehler K. (2019). Anticipatory grasping control modulates somatosensory perception. J. Vis. 19, 4–4. 10.1167/19.5.4 31058990

[B76] Wright D. J. Holmes P. Di Russo F. Loporto M. Smith D. (2012). Reduced motor cortex activity during movement preparation following a period of motor skill practice. PLoS ONE 7, e51886. 10.1371/journal.pone.0051886 23251647 PMC3522608

[B77] Yan L.-R. Wu Y.-B. Zeng X.-H. Gao L.-C. (2015). Dysfunctional putamen modulation during bimanual finger-to-thumb movement in patients with Parkinson's disease. Front. Hum. Neurosci. 9:516. 10.3389/fnhum.2015.00516 26483652 PMC4588113

[B78] Yen C.-T. Lu P.-L. (2013). Thalamus and pain. Acta Anaesthesiol. Taiwanica 51, 73–80. 10.1016/j.aat.2013.06.011 23968658

[B79] Yu H. Sternad D. Corcos D. M. Vaillancourt D. E. (2007). Role of hyperactive cerebellum and motor cortex in Parkinson's disease. NeuroImage. 3, 222–233. 10.1016/j.neuroimage.2006.11.047 17223579 PMC1853309

[B80] Yu X. J. He H. J. Zhang Q. W. Zhao F. Zee C. S. Zhang S. Z. . (2014). Somatotopic reorganization of hand representation in bilateral arm amputees with or without special foot movement skill. Brain. Res. 1546, 9–17. 10.1016/j.brainres.2013.12.025 24373804

[B81] Zaheer A. Malik A. N. Masood T. Fatima S. (2021). Effects of phantom exercises on pain, mobility, and quality of life among lower limb amputees; a randomized controlled trial. BMC Neurol. 21, 416. 10.1186/s12883-021-02441-z 34706654 PMC8554869

[B82] Zeidan F. Lobanov O. V. Kraft R. A. Coghill R. C. (2015). Brain mechanisms supporting violated expectations of pain. Pain 156, 1772–1785. 10.1097/j.pain.0000000000000231 26083664 PMC4545679

[B83] Zhao Y. Zheng X. Wang Q. Xu J. Xu X. Zhang M. (2014). Altered activation in visual cortex: Unusual functional magnetic resonance imaging finding in early Parkinson's disease. J. Int. Med. Res. 42, 503–515. 10.1177/0300060513507647 24595154

[B84] Zheng B.-X. Yin Y. Xiao H. Lui S. Wen C.-B. Dai Y.-E. . (2021). Altered cortical reorganization and brain functional connectivity in phantom limb pain: a functional mri study. Pain Pract. 21, 394–403. 10.1111/papr.12966 33202107

